# Microbes in the Anthropocene: spillover of agriculturally selected bacteria and their impact on natural ecosystems

**DOI:** 10.1098/rspb.2016.0896

**Published:** 2016-12-14

**Authors:** Thomas Bell, Jason M. Tylianakis

**Affiliations:** 1 Department of Life Sciences, Imperial College London, Silwood Park Campus, Buckhurst Road, Ascot, Berkshire SL5 7PY, UK; 2 Centre for Integrative Ecology, School of Biological Sciences, University of Canterbury, Private Bag 4800, Christchurch 8140, New Zealand

**Keywords:** microbial dispersal, agriculture, soil bacteria, source–sink models, ecosystem functioning, local adaptation

## Abstract

Soil microbial communities are enormously diverse, with at least millions of species and trillions of genes unknown to science or poorly described. Soil microbial communities are key components of agriculture, for example, in provisioning nitrogen and protecting crops from pathogens, providing overall ecosystem services in excess of $1000bn per year. It is important to know how humans are affecting this hidden diversity. Much is known about the negative consequences of agricultural intensification on higher organisms, but almost nothing is known about how alterations to landscapes affect microbial diversity, distributions and processes. We review what is known about spatial flows of microbes and their response to land-use change, and outline nine hypotheses to advance research of microbiomes across landscapes. We hypothesize that intensified agriculture selects for certain taxa and genes, which then ‘spill over’ into adjacent unmodified areas and generate a halo of genetic differentiation around agricultural fields. Consequently, the spatial configuration and management intensity of different habitats combines with the dispersal ability of individual taxa to determine the extent of spillover, which can impact the functioning of adjacent unmodified habitats. When landscapes are heterogeneous and dispersal rates are high, this will select for large genomes that allow exploitation of multiple habitats, a process that may be accelerated through horizontal gene transfer. Continued expansion of agriculture will increase genotypic similarity, making microbial community functioning increasingly variable in human-dominated landscapes, potentially also impacting the consistent provisioning of ecosystem services. While the resulting economic costs have not been calculated, it is clear that dispersal dynamics of microbes should be taken into consideration to ensure that ecosystem functioning and services are maintained in agri-ecosystem mosaics.

## 1. Introduction

Agriculture now dominates landscapes across whole continents, and humans are intensifying food and resource production to fuel a growing population [1,2] This land-use intensification is the most important driver of global biodiversity decline [2] by eliminating habitat and selecting for species pre-adapted to agricultural environments. The organisms that succeed and dominate in agriculture, when fertilizers, lime and chemical control methods are used, are not a random subset of those in the surrounding landscape, but are selected by the environment according to their traits. This typically results in one or a few species dominating communities [3], for which agriculture provides abundant resources once environmental constraints have been overcome. If the traits that determine species responses to the environment are correlated with their contribution to ecosystem functioning [4,5], then selection for a subset of species can cause rapid loss of functional diversity [6] with potential impacts on ecosystem services and resilience [7]. At larger scales, agricultural intensification also alters beta diversity (the change in species composition across locations) by eliminating disturbance-sensitive species and reducing the natural variety of habitats [8,9], which has the potential to impact the resilience of ecosystem services to perturbations such as climate change.

Substantial prior ecological research has examined how changes to local communities and populations can impact landscape-level properties [10] such as the dispersal of fishes from marine reserves into surrounding fished areas [11]. Likewise, recent evidence has shown that insect predators associated with agriculture can attain high abundance owing to high agricultural productivity, then spill over into adjacent environments where they can exert predation pressure on native species [12]. The common theme is that anthropogenic alterations to the environment have selected for particular traits or species, and those have then had consequences for community dynamics and ecosystem functioning at larger spatial scales owing to species movement.

The effects of agricultural intensification on ecological communities are most evident in macroscopic organisms. For example, conversion of forest to agriculture impacts vertebrate [13] and invertebrate communities [12] both above- and below-ground [14]. The physical and chemical properties of soils typically change following conversion to agriculture, so microscopic organisms probably also respond to agriculture in important ways. For example, nutrient addition experiments across continents generated consistent effects on bacterial community composition (though not functional diversity) [15]. Agricultural soils typically contain more available nutrients, owing to inputs of fertilizer and carbon, because crops tend to be fast-growing plant species with low lignin content, which can influence microbial composition [16]. Changes to soil conditions can alter microbial communities in ways that are correlated with changes to key soil ecosystem properties [17], such as their stability in the face of drought [18] or their ability to sequester carbon [19]. There is even evidence that agricultural regimes can select for specific microbial communities across large spatial scales, for example across the Amazon basin [20].

Despite this growing evidence that agricultural intensification impacts soil microbes, the importance of landscape processes (such as changes in beta diversity, spillover and trait filtering; e.g. [10]) has not been studied in detail in microbial communities. Microbial communities are extraordinarily complex and diverse, with thousands of taxa occupying every gram of soil. While it is unclear how much of this diversity is living or active [21], there is a long-held assumption that, owing to this enormous diversity, microbes are so abundant and so cosmopolitan that dispersal limitation is unimportant [22]. In the absence of dispersal limitation, there would be little need to incorporate landscape-level processes to understand local communities. While this assumption of global dispersal persisted for a long period, it is now clear that microbes exhibit distinct spatial patterns, from scales of micrometres [23] to continents [24]. Even with state-of-the-art sequencing, it is not possible to obtain comprehensive surveys of all individuals within a soil microbial community [25]. Nonetheless, there are clear geographical patterns in natural environments for the dominant taxa, and well-known spatial patterns for strains of medical or agricultural interest [26]. It is therefore clear that understanding microbial communities requires knowledge of how microbial taxa disperse across landscapes, and how dispersing individuals are able to colonize novel environments, alter recipient communities and impact ecosystem functioning and services.

Agriculture has had negative impacts on aboveground biodiversity, probably owing to the combined effects of agricultural policy and farming practice on environmental heterogeneity [27]. Here, we discuss some potential consequences of agricultural intensification for soil microbial communities at landscape scales. Although we focus on the bacterial component of soil communities, the processes we discuss are likely to be more broadly applicable to microbial communities in general that are responding to large-scale disturbance (e.g. sewage works flowing into rivers, marine fish farms, responses to global nitrogen deposition, etc.). Specifically, we discuss the potential for landscape processes to shape microbial communities and functional processes in any location, and the potential role of dispersal and landscape structure in mediating these effects. Our discussion is structured around nine testable hypotheses (summarized in the electronic supplementary material, table S1), which together provide a potential platform to understand spatially structured microbial communities, and as a way to establish common goals for future research in the area.

## 2. Landscape-level impacts of agriculture on microbial communities

*Hypothesis 1*. Agricultural intensification selects for taxa and genotypes, which may differ from those in natural environments.

Agricultural practices vary widely depending on local conditions and crops. We focus here on intensive agriculture, defined broadly as land-use modifications for agriculture resulting in alterations to above- or below-ground environmental conditions. Using such a broad definition, we do not expect a single response of soil microbial communities to agricultural intensification *per se* because farming practices will vary from farm to farm, probably leading to a complex selection mosaic. However, several studies have focused on individual components of agricultural intensification and found impacts on microbial community composition. Surveys and experiments have suggested that soil pH is the most important factor governing the diversity and composition of soil bacterial communities [28,29], and probably other soil microbial taxa [28]. Though less studied, many other common agricultural practices have also shown strong impacts on soil bacterial communities. For example, Fierer *et al*. [30] recently demonstrated that nitrogen fertilization altered within-habitat bacterial community composition, the abundance of genes involved in key subsystems, and the community catabolic ability. Similar effects have been observed under experimental and field addition of herbicide [31] because target enzymes and pathways of insecticides or herbicides may also be present in microbes.

**Figure 1.**
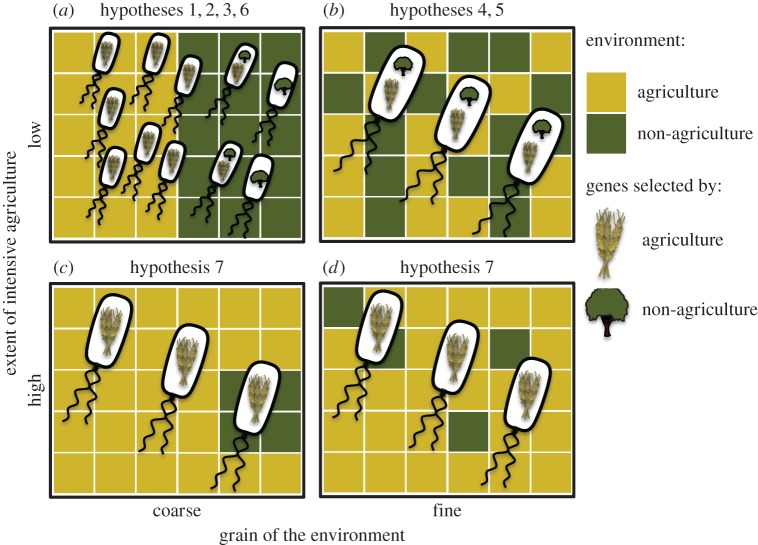
Illustration of some of the hypotheses discussed in the main text. Each panel is a landscape, with different colours indicating different habitat types (agriculture or no agriculture). The landscapes are arranged to illustrate coarse-grained (habitat patch size much larger than dispersal range, left column) and fine-grained (habitat patch size similar to- or smaller than dispersal range, right column) landscapes, as well as landscapes more (bottom row) or less (top row) dominated by intensive agriculture. Bacterial cells are placed across the landscape and the symbol inside represents the habitats to which they are best adapted. (*a*) Ecotypes are locally adapted to agriculture and non-agriculture environments, as described in hypothesis 1. Owing to the extent of the area under agricultural intensification, genes or species spill over into adjacent non-agriculture areas (hypothesis 2), creating a halo of niche differentiation either of agriculture-adapted strains (hypothesis 3) or introgression of genes selected under agriculture (hypothesis 6). (*b*) Finer-grained environments select for generalist strains, with adaptations to both agriculture and non-agricultural environments, because both environments are encountered (hypothesis 5). Equivalently, strains with higher dispersal abilities will select for generalist species (hypothesis 4). (*c*) Increasing the extent of intensive agriculture will result in landscapes dominated by agriculture specialists, because spillover from agriculture swamps locally adapted strains in non-agriculture environments, resulting in a loss of beta diversity (hypothesis 7). (*d*) The grain of the environment has a weaker effect on bacterial populations when the extent of intensive agriculture is high.

Intensive agriculture not only imposes a filter, selecting from the available genetic diversity, but it may also drive the evolution of new traits. For example, the growing use of antibiotics, pesticides and herbicides in agriculture [32] has driven the rapid evolution of resistant microbes, in addition to arthropod pests and weeds [33,34]. Consequently, populations of microbes may form ‘ecotypes’ with genotypic differences specific to their local environment [35,36] (figure 1*a*). Development of ecotypes that specialize on local conditions has been demonstrated under laboratory conditions [37], and also at large spatial scales in natural environments [35,38]. While there has not been evidence of ecotypes with specific adaptations to agriculture, we speculate that such ecotypes exist. Indeed, there is ample evidence of bacterial and fungal pathovars associated with specific crops. The substantial impact of agriculture on a wide range of abiotic factors in the soil environment, combined with the laboratory and field studies described above, suggest that strong selective pressures will not only operate ecologically to sort species, but will probably also drive diversification of individual taxa into ecotypes that might be distinct from surrounding areas.

Multicellular organisms, such as arthropods and weeds, have evolved adaptations to agricultural conditions within a few generations [34,39]. However, microbial communities may evolve responses to agriculture on a much larger scale, for reasons beyond simply their short generation times. Specifically, the ability of microbes to pass genetic material horizontally across taxa (even across domains of life [40]), either via mobile genetic elements or by uptake of naked DNA from the environment, could allow the selection for traits across a whole community, rather than just within individual taxa. In this way, entire metabolic pathways (e.g. nitrogen fixation, xenobiotic degradation or pathogenicity [41]) can be transferred between microbes via genomic islands [42]. Likewise, the tetracycline resistance gene *tet*M has been detected among typical soil inhabitants downstream from pig farms [43], suggesting horizontal transfer. Although the frequency with which such genes are horizontally transmitted in nature is not clear [41], large genomic similarities across unrelated taxa [35] suggest that it can be an important process in some instances. The mechanisms therefore clearly exist for traits to rapidly evolve and then spread across taxa when humans create intensive selection pressures.

Overall, current evidence suggests that a range of ecological and evolutionary processes mediate the impact of intensive agriculture on the fitness of taxa and genotypes. In what follows, we hypothesize that these alterations within agriculture have the potential to spill over into natural ecosystems to create changes across whole landscapes, with potential impacts on ecosystem functioning, services and resilience.

*Hypothesis 2*. Agriculturally selected genes will spill over into adjacent unmodified ecosystems.

Spatial flows of organisms are known to be crucial for altering communities and maintaining populations [44,45]. Many studies have focused on the flow of organisms between disturbed and undisturbed sites across a landscape. Yet despite a disproportionate amount of research examining flows of beneficial organisms from natural into managed habitats [46], both nutrients [47] and organisms (such as predators, pathogens and pollinators [48,49]) can also flow in the opposite direction. While this process is fairly well studied in plants and animals [48,49], particularly in species with high dispersal range, such as flying insects [49], it remains poorly understood in passively dispersing organisms, such as microbes. More importantly, given the potential for agriculture to drive genetic changes in microbial populations (described above), it is unclear whether genes selected in one habitat spread across edges into adjacent habitats. Empirical work has confirmed the potential for bacterial cells to disperse from agricultural fields [50]. While the number of dispersing cells is small relative to the numbers found within the soil, such dispersal events provide the opportunity for genetic spillover. In combination with the capacity for horizontal gene transfer (HGT) among many soil microbes, agricultural practices therefore have the capacity to have a large impact on the genetic structure and functioning of microbial communities in adjacent unmodified habitats.

Widespread intensive agriculture therefore provides an opportunity for genes (and their corresponding phenotypic traits), which are selected in agriculture to impinge on the surrounding communities (figure 1*a*). Species with traits selected in agricultural areas can thus move into, and potentially alter, the ecology of surrounding habitats affecting their functioning and capacity to provide services [49]. For example, it is feasible that bacteria that are resistant to pesticides and antibiotics [34,39] will drive ‘genetic spillover’ into adjacent natural habitats, thereby altering competitive dynamics when aerial agrichemical drift also impacts the surroundings. In cases where the high productivity of agriculture supports more rapid population growth, spillover from agriculture may be greater than in the opposite direction (as observed in invertebrates [12]).

Even if microbial taxa do spill over from agriculture, there are no guarantees of an ecological impact. Controlled releases of genetically modified bacteria indicate they decline rapidly and often fail to disperse outside the release sites [51]. In addition, although soil and airborne microbial composition has been shown to differ across land-use and plant-cover types [52,53], spillover from managed to natural habitats is not documented for soil microbial communities. Nevertheless, we hypothesize that the frequency and ecological impact of spillover processes will depend on the intensity, spatial extent and spatial patterning of agriculture in the landscape. This patterning will also determine relative fitness differences among subpopulations (or genotypes), which we expect will determine the subsequent introgression of agriculturally selected genes into the surrounding landscape. Therefore, we will now discuss how landscape structure may determine the impacts of spillover processes (hypothesis 3) and the role of dispersal in determining outcomes (hypothesis 4).

*Hypothesis 3*. Landscape composition will mediate the impact of genetic spillover.

Spillover of organisms and their genes from agriculture to surrounding areas can be understood in terms of source–sink models [54]. Source populations are defined as populations that experience environmental conditions which allow them to maintain positive growth rates over the long term. Net positive growth allows the population to expand into the available space and to produce emigrants that can colonize adjacent areas (see hypotheses 1 and 2). Sink populations are defined as populations with net negative growth rates over the long term because environmental conditions are poor from the perspective of the population. Such populations would become extinct in isolation, but can persist over the long term if they are maintained by active or passive immigration [54,55]. If the flow of individuals from agricultural to natural habitats is asymmetric (i.e. there is net spillover from agriculture, hypothesis 2), this could in theory maintain populations with agriculturally selected traits in adjacent environments, even if the novel traits are maladapted [56] (figure 1*a*).

Source–sink models have demonstrated how studies of habitats in isolation can produce misleading conclusions. Populations that are outside of their fundamental niche, with low capacity for adaptation owing to small population sizes, can be sustained for long periods by source communities, which act as generators of genetic diversity. In this context, areas experiencing intensified agriculture can be viewed as source communities for a wide range of populations that are adapted to the regime of high disturbance and nutrient availability associated with intensive agriculture. Conversely, the initial colonization of agriculture by new variants can be viewed as a constant probing by the surrounding species pool to find a genetic variant that is pre-adapted to the agricultural sink community. Once that variant is found, it is able to achieve positive growth in the sink community (agriculture), which then becomes a source community. Rates of evolution will be most rapid when population sizes are large, and large effective population sizes can also be achieved when movement couples non-contiguous populations [57]. Combined with the high potential for population growth afforded by the nutrient-rich conditions in intensified agriculture, we expect rapid evolutionary change (e.g. adaptation to pesticides or other stressors) within agricultural habitats (hypotheses 1 and 2). Emigration of successful variants from highly productive agricultural habitats subsequently has the potential to constrain adaptive evolution in the surrounding landscape by flooding it with variants that are maladapted outside of their agricultural context. In addition, many soil bacteria can prolong survival through the creation of stress-resistant spores [58] or transform to a dormant state [58]. Such a strategy could result in the build-up of dormant agriculture-derived cells in areas adjacent to agriculture, which would be available to exploit any conditions that become favourable. Source–sink dynamics could be important for bacterial communities in principle, but have rarely been demonstrated in nature. Examples do exist, but are largely restricted to simplified microcosm systems [59].

If source–sink dynamics are common in microbial systems, we expect the extent to which agriculturally selected traits become fixed across the landscape will depend, for each taxon, on the proportion of its population in source versus sink habitats [56]. However, because farming practices will vary from farm to farm, the ‘proportion of land under intensification’ is likely to be complex. Evolutionary dynamics in heterogeneous landscapes will depend on their fitness across the landscape and the dispersal ability of each taxon [60,61]. For an environment that varies over space, the variation in environmental conditions experienced by a population will depend on the dispersal rate of the organism; we expect that the range over which spillover can drive introgression of genes into sink habitats of the surrounding landscape will depend on the interplay between dispersal range (hypothesis 4) and the distance between source patches in the landscape [57]. There is thus significant theoretical support for the hypothesis that the magnitude and effects of spillover will depend on the amount of agriculture, its intensity in each patch and the spatial configuration of those patches within the landscape.

The above discussion of source–sink dynamics has assumed that fitness benefits in agriculture are traded off against fitness costs outside of the agricultural environment (or under different agricultural management regimes), and thus that agriculturally selected genotypes are maintained in the wider landscape by immigration alone. However, it is also possible that, once selected by agriculture, a new variant will experience ‘unforeseen’ fitness benefits (i.e. ‘positive pleiotropy’) in different contexts [62]. As with the source–sink processes above, the importance of this process will depend on landscape heterogeneity, in particular the extent of cross-habitat differences in selective regime.

*Hypothesis 4*. The rate and range of dispersal will determine the extent of genetic spillover.

Source–sink dynamics depend on the interplay between dispersal ability and landscape composition. If there is low dispersal among patches that differ in their environmental conditions, and if the phenotypes favoured in the different habitats differ strongly, selection will favour specialization of organisms to the habitat in which fitness, and hence population size, was initially highest [56]. However, as dispersal among patches increases, selection drives equalization of the fitness in both populations (i.e. increased habitat generalism) [56,63], particularly if dispersal occurs in both directions (in to- and out of- areas of intensive agriculture) and is passive or density-independent [64]. Coexistence of subpopulations and maintenance of regional genetic and species diversity is threatened by high dispersal rates [57], such that selection for increased generalism comes at the expense of habitat specialization. Dispersal range relative to the size of habitat patches (i.e. the grain of the environment) therefore plays a key role in determining the role of landscape-level processes (figure 1). If dispersal is too high, we expect homogeneous populations of generalists across the landscape. If dispersal is too low, there is little opportunity for maladapted migrants to play a role because they only rarely encounter foreign environments. If there is a high rate and range of dispersal, dispersal limitation may be important for structuring bacterial communities, but only over short timescales [65]. Under this scenario, we would expect the importance of landscape processes to shift over time depending on the frequency with which farming practices change.

The ability of genotypes or genes to become fixed regionally through the processes outlined in hypotheses 1–3 will therefore depend on dispersal dynamics. There is a great deficiency of estimates of dispersal rates in bacteria. While there is some information on the quantity of airborne bacteria and other microbes, and evidence of the potential for long-distance dispersal in dust [66], there are few if any quantifications of dispersal rates with the exception of a few (mostly pathogenic) taxa.

Finally, if source–sink dynamics are important for most bacterial populations within a community, we expect a decline both in the abundance of source bacterial populations, and of overall community similarity (i.e. increasing beta diversity) with distance from a source. The rate of decay should depend on the dispersal ability of the taxon, and should become steeper in heterogeneous landscapes (where the probability declines of having similar selection pressures inside and outside the source habitat), as observed in marine habitats [67]. We therefore hypothesize that spillover effects will generate a ‘halo’ of genetic differentiation of each taxon (see hypothesis 6) around the habitat in which selection occurred, which will fade in more distant populations (figure 1).

*Hypothesis 5*. There will be selection for large genomes when landscapes are heterogeneous and dispersal rates are high—the ‘Swiss-army genome’ hypothesis.

Bacterial genomes tend to be small and contain few non-coding regions relative to eukaryotes [68]. Also unlike eukaryotes, genome size is strongly related to the number of functional genes, making genome size an important topic of study in bacteria [69]. All other things being equal, this pattern would suggest that bacteria with larger genomes have a greater functional repertoire, and can therefore more readily adapt to diverse environments [70]. Congruent with this hypothesis, surveys have revealed that taxa which carried a suite of metabolic pathways relating to xenobiotic degradation were more ubiquitous, possibly because they could withstand a greater variety of local stressors [71]. However, expression of large genomes is energetically costly [72], so bacterial genome size will be reduced in the absence of strong selection [73].

If the landscape-scale processes hypothesized above (hypotheses 2–4) occur frequently, there is the opportunity both for the horizontal transfer of genetic material and strong selection pressures needed to maintain large genomes in the landscape. Taxa with a large genetic repertoire may be expected to perform better in more heterogeneous landscapes, or when conditions are highly variable temporally (e.g. owing to harvesting or timing of chemical inputs) (figure 1*b*). This would be an extension of the processes discussed in hypothesis 4, because the evolution of cross-environment generalism (outweighing habitat-specific specialization when dispersal rates are high and habitats are heterogeneous) could occur through selection of larger genomes, and taxa with initially large genomes may be those most able to survive initially following the introduction of agriculture to the landscape (hypothesis 1).

By contrast, within agricultural land itself, selection will probably favour a specific set of phenotypic traits rather than genetic plasticity. For example, the addition of nutrients has recently been shown to reduce the effective genome size of soil bacteria across several continents and soil types [15] congruent with the idea that large genomes are advantageous in nutrient-limited environments where resources are scarce but diverse [69]. In this sense, increased genome size would be selected for in heterogeneous landscapes with some intensified areas. However, if intensification spreads to comprise the entire landscape, the benefit of a large genetic repertoire would be expected to decline, and we would expect to see reduced genome size.

## 3. From process to pattern

We have discussed ways in which landscape processes might influence populations and genotypes in landscapes subjected to intensive agriculture. In what follows, we discuss some of the patterns that we would expect to observe if landscape processes are important.

*Hypothesis 6*. There will be a halo of genetic differentiation surrounding intensive agriculture.

The constant movement of agriculture-derived bacterial cells into adjacent areas (figure 1*a*) can generate several different outcomes. Perhaps, the most likely is that the cells will rapidly die off. As a null hypothesis, agricultural soils might represent drastically different environmental conditions relative to adjacent areas, so emigrating cells are unlikely to be pre-adapted to these new conditions. Under this scenario, the standing stock of emigrated cells in the surroundings will simply reflect the balance between the emigration rate and the mortality rate, resulting in a halo of dead and dying cells around the agricultural area. The persistence time of DNA would probably make this halo detectable with genomic approaches, but their inactivity would render them unlikely to appear important in transcriptomic (i.e. transcribed RNA representing active genes) surveys. However, there are also other likely scenarios.

First, the emigrating bacterial subpopulation might remain active, but experience negative population growth. For example, many studies have detected elevated levels of antibiotic resistance genes within sewage treatment plants and in the surrounding environment [74]. There is a rapid decline of antibiotic resistance genes with distance from the treatment plants, indicating a cost to carriage of the genes that is difficult to detect under laboratory conditions, resulting in negative population growth rates in low-antibiotic environments. Similarly, there are rapid rates of decline of well-studied obligate pathogens when dispersing into natural environments [75]. While decline rates are rapid, they are by no means instantaneous even for bacteria for which soil must be extremely harsh relative to their typical environment. Declining subpopulations can theoretically be ‘rescued’ from local extinction by the source–sink dynamics described above. While the population growth rate might be exceeded by the mortality rate to yield a net negative growth, the population will still continue to evolve as it turns over. If it evolves sufficiently rapidly, there is the potential for the population to achieve positive growth rates. Evolutionary rescue has received increasing attention in the literature, and has been demonstrated for bacteria in simplified ecosystems [76]. Over the short term, there would be a halo of populations experiencing negative population growth, and the halo would increase in diameter as populations are rescued.

Second, there is the potential to confer genetic material via HGT (see discussion in hypothesis 1). Under this scenario, genes could be treated in the same way as the above: genes that are at low abundance in sink communities could be rescued via HGT. Here, it is not the dispersal of individuals that is important, but rather the continual renewal of genetic material that is available for HGT to the resident community. In this way, HGT can have important impacts on ecosystem services, notably in providing the machinery for nitrogen cycling in many lineages, and also for pathogenicity in *Pseudomonas syringae* [77]. Although the size of the halo, the extent of its genetic differentiation and the rate of evolution within it should differ across taxa, greater prevalence of HGT would be expected to increase the correlation in response across taxa.

Finally, traits selected in agriculture may provide unexpected benefits in the surrounding environment. In these cases, the halo of genetic differentiation would continue to spread across the landscape, limited primarily by the dispersal rate of the taxa carrying these genes.

*Hypothesis 7*. Landscape simplification will drive genetic homogenization both within and across taxa.

Consistent selection by agriculture for specific taxa and traits (hypothesis 1) has been shown in insects to reduce the site-to-site dissimilarity in species composition (beta diversity) relative to more heterogeneous natural habitats [8]. Findings that agricultural practices such as application of limestone can have consistent effects on microbial communities across different locations [28], and thus be associated with an increased homogenization (lower beta diversity) of soil bacterial communities at a landscape level [20], suggest that microbes may also exhibit lower beta diversity across agricultural habitats compared with natural habitats. As the proportion of agriculture in the landscape increases, we would expect increased similarity in species composition across the landscape (figure 1*c*,*d*).

In addition to selecting for certain taxa, studies on multicellular organisms suggest that agriculture selects for taxa that are functionally similar [5,6], which might also occur for microbial community structure [20] and function [78]. In microbes, this increasing functional similarity could partly entail higher genetic similarity within and across taxa, via selection for a subset of the community with shared vertically inherited or horizontally transmitted genes. In addition, high rates of spillover (hypothesis 2), any evolutionary tendency towards habitat generalism (hypothesis 4) or any additional benefits of agriculturally selected traits outside the originally selected environment (i.e. positive pleiotropy, see hypothesis 3) would mean that agriculturally selected subpopulations could survive, and could have a competitive advantage over ancestral subpopulations of the same taxon in surrounding habitats. This would eventually replace diverse, habitat-specific ecotypes [35,36] of each taxon across the landscape with a single ecotype that thrives in agriculture. Therefore, we hypothesize that agricultural intensification will drive homogenization of the microbial genetic landscape (and an overall reduction in landscape genetic diversity) by increasing between- and within-taxon genetic similarity, in addition to driving taxonomic homogenization.

## 4. Consequences of spillover for ecosystem functioning

*Hypothesis 8*. The effects of spillover on community and genotypic composition will alter ecosystem functioning.

Laboratory experiments have shown a direct relationship between microbial community structure and function [79]. The relationship in natural environments has been more mixed, with field manipulations and correlational studies of microbial communities showing a range of responses [80]. However, large-scale surveys across land-use types in Europe have shown significant relationships between the microbial component of soils and soil functioning across a broad array of functions [17]. It is therefore plausible that spillover can alter ecosystem functioning. One issue is that field estimates of community structure only capture a small percentage of the community. This might be particularly problematic if rare species contribute significantly to functional processes, as is the case in some instances [81]. In the context of spillover from agriculture, strains that are initially rare would pass unnoted in the long tail of rare species, only coming to the fore once they alter functional processes or become more abundant following a period of adaptation.

Another key distinction is whether the functional changes resulting from processes such as hypotheses 6 and 7 occur because of changes in taxonomic composition or owing to genetic changes within taxa (e.g. HGT). The former will result from community changes that are detectable with amplicon sequencing, and can encompass biochemically complex traits that are strongly phylogenetically conserved (e.g. pH and salinity) [82]. The latter pathway to functional change appears to be less prevalent across the microbial tree of life, but can still allow rapid phenotypic changes (e.g. antibiotic resistance, organic phosphorus uptake or use of simple carbon substrates) in ways that are not explained by changes to taxonomic composition [82].

*Hypothesis 9*. Microbial processes across the landscape will become increasingly variable owing to loss of genetic insurance against environmental change/global stressors.

The homogenization of microbial community genetics caused by agricultural practices (hypothesis 7) represents a loss of genetic ‘insurance’ [9], which could have important consequences for the stability of ecosystem functioning at the landscape scale [83]. Although few empirical examples of this phenomenon exist (e.g. [17]), there is strong theoretical support for this ‘insurance’ hypothesis [84]. This hypothesis posits that asynchronous responses of different species or phenotypes to environmental fluctuations may stabilize aggregate ecosystem processes by ‘averaging out’ individual responses. Although originally proposed as an extension of biodiversity effects through time [84], similar insurance effects can occur spatially [9]. If habitats exhibit asynchronous fluctuations (e.g. because of harvesting times or different growth rates), spatial averaging could increase aggregated measures of functioning across a landscape. In a heterogeneous landscape, dispersal of species among habitats allows rapid recolonization of a habitat following disturbance. In addition, if the landscape contains a large variety of locally adapted species, these habitat specialists can be drawn upon as conditions change across the landscape [9]. Metacommunity models, which model the dynamics of isolated communities connected by dispersal, have shown that spatial insurance effects maximize biodiversity and ecosystem functioning (while minimizing variability in functioning) when dispersal rate is intermediate. Similarly, functioning is maximized and its variability minimized when diversity is high [9].

We summarize the predicted outcomes of the hypotheses in figure 1. The processes we hypothesize above would affect these spatial insurance effects in four important ways. First, the environments imposed by agriculture will select for certain taxa, which will reduce the taxonomic and genetic diversity in agricultural habitats (lower alpha diversity; hypothesis 1). Second, spillover and source–sink dynamics will also reduce between-habitat differences in taxonomic and genetic composition (lower beta diversity; hypothesis 7). Third, as intensification proceeds, these two processes will reduce the total diversity of the landscape (lower gamma diversity). Fourth, in heterogeneous landscapes, the environmental correlation among habitats is lowest, but the expansion of intensive agriculture will generate an increasingly spatially synchronized environment. These four processes have recently been shown to collectively drive insurance effects in metacommunity models [85].

## 5. Prospects

Modern agriculture represents an important landscape modification that has far-reaching impacts on the taxonomic and genetic structure and function of soil microbial communities. There is often an assumption that microbial community dynamics are local and can be understood without reference to the wider landscape. Whether this is true depends critically on the scale at which ecological and evolutionary pressures imposed by agricultural intensification operate. Our review supports there being landscape-scale impacts of agriculture on the ecology and evolution of microbial communities; the importance of landscape-scale processes will therefore depend on dispersal rates and on the ability of dispersing cells to colonize habitats outside of agriculture, which will in turn interact with the extent and patterning of agriculture across the landscape. There is clearly a constant rain of dispersing microbial cells in the air and through the soil, but estimates of dispersal and colonization rates are virtually unknown for microbes except for a few plant and animal pathogens. We have laid out a series of testable hypotheses and their predictions for placing the impacts of agriculture on microbial populations within an agricultural context, but most hypothesis tests rely on an understanding of the rate and fate of dispersing cells. However, tracking dispersing microbial populations in the wild remains a logistical challenge. Rapid improvements in sequencing technology are likely to provide solutions either for targeted or abundant populations. Further advances are likely to come from new technologies or new experiments that can track microbes across landscapes. If these logistical challenges can be overcome, and the relationship between microbial community structure and functioning can be better resolved, we could potentially achieve the understanding needed to inform the management of agricultural landscapes with a focus on preserving the crucial functions performed by microbial communities.

## Supplementary Material

Table S1: Summary of the hypotheses

## References

[1] Tilman D, Clark M 2014 Global diets link environmental sustainability and human health. Nature 515, 518–522. (10.1038/nature13959) 25383533

[2] Newbold T et al. 2016 Has land use pushed terrestrial biodiversity beyond the planetary boundary? A global assessment. Science 353, 288–291. (10.1126/science.aaf2201) 27418509

[3] Stevens CJ, Dise NB, Mountford JO, Gowing DJ 2004 Impact of nitrogen deposition on the species richness of grasslands. Science 303, 1876–1879. (10.1126/science.1094678) 15031507

[4] Lavorel S, Garnier E 2002 Predicting changes in community composition and ecosystem functioning from plant traits: revisting the Holy Grail. Funct. Ecol. 16, 545–556. (10.1046/J.1365-2435.2002.00664.X)

[5] Larsen TH, Williams NM, Kremen C 2005 Extinction order and altered community structure rapidly disrupt ecosystem functioning. Ecol. Lett. 8, 538–547. (10.1111/j.1461-0248.2005.00749.x) 21352458

[6] Rader R, Bartomeus I, Tylianakis JM, Laliberté E 2014 The winners and losers of land use intensification: pollinator community disassembly is non-random and alters functional diversity. Divers. Distrib. 20, 908–917. (10.1111/ddi.12221)

[7] Cadotte MW, Carscadden K, Mirotchnick N 2011 Beyond species: functional diversity and the maintenance of ecological processes and services. J. Appl. Ecol. 48, 1079–1087. (10.1111/j.1365-2664.2011.02048.x)

[8] Tylianakis JM, Klein A-M, Tscharntke T 2005 Spatiotemporal variation in the diversity of Hymenoptera across a tropical habitat gradient. Ecology 86, 3296–3302. (10.1890/05-0371) 17249230

[9] Loreau M, Mouquet N, Gonzalez A 2003 Biodiversity as spatial insurance in heterogeneous landscapes. Proc. Natl Acad. Sci. USA 100, 12 765–12 770. (10.1073/pnas.2235465100) PMC240692 14569008

[10] Tscharntke T et al. 2012 Landscape moderation of biodiversity patterns and processes: eight hypotheses. Biol. Rev. 87, 661–685. (10.1111/j.1469-185X.2011.00216.x) 22272640

[11] Halpern BS, Lester SE, Kellner JB 2009 Spillover from marine reserves and the replenishment of fished stocks. Environ. Conserv. 36, 268–276. (10.1017/S0376892910000032)

[12] Frost CM, Didham RK, Rand TA, Peralta G, Tylianakis JM 2015 Community-level net spillover of natural enemies from managed to natural forest. Ecology 96, 193–202. (10.1890/14-0696.1) 26236904

[13] Donald PF, Sanderson FJ, Burfield IJ, van Bommel FPJ 2006 Further evidence of continent-wide impacts of agricultural intensification on European farmland birds, 1990–2000. Agric. Ecosyst. Environ. 116, 189–196. (10.1016/j.agee.2006.02.007)

[14] Wolters V et al. 2000 Effects of global changes on above- and belowground biodiversity in terrestrial ecosystems: implications for ecosystem functioning. Bioscience 50, 1089–1098. 10.1641/0006-3568(2000)050%5B1089:EOGCOA%5D2.0.CO;2)

[15] Leff JW et al. 2015 Consistent responses of soil microbial communities to elevated nutrient inputs in grasslands across the globe. Proc. Natl Acad. Sci. USA 112, 10 967–10 972. (10.1073/pnas.1508382112) 26283343 PMC4568213

[16] Lauber CL, Strickland MS, Bradford MA, Fierer N 2008 The influence of soil properties on the structure of bacterial and fungal communities across land-use types. Soil Biol. Biochem. 40, 2407–2415. (10.1016/j.soilbio.2008.05.021)

[17] de Vries FT et al. 2013 Soil food web properties explain ecosystem services across European land use systems. Proc. Natl Acad. Sci. USA 110, 14 296–14 301. (10.1073/pnas.1305198110) PMC3761618 23940339

[18] de Vries F, Liiri ME, Bjørnlund L, Bowker MA. Christensen S, Setälä HM, Bardgett RD 2012 Land use alters the resistance and resilience of soil food webs to drought. Nat. Clim. Chang. 2, 276–280. (10.1038/nclimate1368)

[19] Post WM, Kwon KC 2000 Soil carbon sequestration and land-use change: processes and potential. Glob. Change Biol. 6, 317–327. (10.1046/j.1365-2486.2000.00308.x)

[20] Rodrigues JLM et al. 2013 Conversion of the Amazon rainforest to agriculture results in biotic homogenization of soil bacterial communities. Proc. Natl Acad. Sci. USA 110, 988–993. (10.1073/pnas.1220608110) 23271810 PMC3549139

[21] Carini P, Marsden PJ, Leff JW, Morgan EE, Strickland MS, Fierer N 2016 Relic DNA is abundant in soil and obscures estimates of soil microbial diversity. bioRxiv. (10.1101/043372) 27991881

[22] Fenchel T, Finlay BJ 2004 The ubiquity of small species: patterns of local and global diversity. Bioscience 54, 777–784. (10.1641/0006-3568(2004)054%5B0777:TUOSSP%5D2.0.CO;2)

[23] Vos M, Wolf AB, Jennings SJ, Kowalchuk GA 2013 Micro-scale determinants of bacterial diversity in soil. FEMS Microbiol. Rev. 37, 936–954. (10.1111/1574-6976.12023) 23550883

[24] Hanson CA, Fuhrman JA, Horner-Devine MC, Martiny JBH 2012 Beyond biogeographic patterns: processes shaping the microbial landscape. Nat. Rev. Microbiol. 10, 497–506. (10.1038/nrmicro2795) 22580365

[25] Quince C, Curtis TP, Sloan WT 2008 The rational exploration of microbial diversity. ISME J. 2, 997–1006. (10.1038/ismej.2008.69) 18650928

[26] Santini A et al. 2013 Biogeographical patterns and determinants of invasion by forest pathogens in Europe. New Phytol. 197, 238–250. (10.1111/j.1469-8137.2012.04364.x) 23057437

[27] Benton TG, Vickery JA, Wilson JD 2003 Farmland biodiversity: is habitat heterogeneity the key? Trends Ecol. Evol. 18, 182–188. (10.1016/S0169-5347(03)00011-9)

[28] Zhalnina K, Dias R, de Quadros PD, Davis-Richardson A, Camargo FAO, Clark IM, McGrath SP, Hirsch PR, Triplett EW 2014 Soil pH determines microbial diversity and composition in the park grass experiment. Microb. Ecol. 69, 395–406. (10.1007/s00248-014-0530-2) 25395291

[29] Griffiths RI, Thomson BC, James P, Bell T, Bailey M, Whiteley AS, Lane B, Gifford C 2011 The bacterial biogeography of British soils. Environ. Microbiol. 13, 1642–1654. (10.1111/j.1462-2920.2011.02480.x) 21507180

[30] Fierer N, Lauber CL, Ramirez KS, Zaneveld J, Bradford MA, Knight R 2012 Comparative metagenomic, phylogenetic and physiological analyses of soil microbial communities across nitrogen gradients. ISME J. 6, 1007–1017. (10.1038/ismej.2011.159) 22134642 PMC3329107

[31] Aguayo P, González C, Barra R, Becerra J, Martínez M 2014 Herbicides induce change in metabolic and genetic diversity of bacterial community from a cold oligotrophic lake. World J. Microbiol. Biotechnol. 30, 1101–1110. (10.1007/s11274-013-1530-y) 24158391

[32] Pimentel D et al. 1992 Environmental and economic cost of pesticide use. Bioscience 42, 750–760. (10.2307/1311994)

[33] Seiler C, Berendonk TU 2012 Heavy metal driven co-selection of antibiotic resistance in soil and water bodies impacted by agriculture and aquaculture. Front. Microbiol. 3, 399 (10.3389/fmicb.2012.00399) 23248620 PMC3522115

[34] Palumbi SR 2001 Humans as the world's greatest evolutionary force. Science 293, 1786–1790. (10.1126/science.293.5536.1786) 11546863

[35] Coleman ML, Chisholm SW 2010 Ecosystem-specific selection pressures revealed through comparative population genomics. Proc. Natl Acad. Sci. USA 107, 18 634–18 639. (10.1073/pnas.1009480107) PMC2972931 20937887

[36] Polz MF, Alm EJ, Hanage WP 2013 Horizontal gene transfer and the evolution of bacterial and archaeal population structure. Trends Genet. 29, 170–175. (10.1016/j.tig.2012.12.006) 23332119 PMC3760709

[37] Buckling A, Kassen R, Bell G, Rainey PPB 2000 Disturbance and diversity in experimental microcosms. Nature 408, 961–964. (10.1038/35050080) 11140680

[38] Cohan FM 2001 Bacterial species and speciation. Syst. Biol. 50, 513–524. (10.1080/10635150118398) 12116650

[39] Khachatourians GG 1998 Agricultural use of antibiotics and the evolution and transfer of antibiotic-resistant bacteria. CMAJ 159, 1129–1136. 9835883 PMC1229782

[40] Nelson KE et al. 1999 Evidence for lateral gene transfer between Archaea and bacteria from genome sequence of *Thermotoga maritima*. Nature 399, 323–329. (10.1038/20601) 10360571

[41] Davison J 1999 Genetic exchange between bacteria in the environment. Plasmid 42, 73–91. (10.1006/plas.1999.1421) 10489325

[42] Sullivan JT, Ronson CW 1998 Evolution of rhizobia by acquisition of a 500-kb symbiosis island that integrates into a phe-tRNA gene. Proc. Natl Acad. Sci. USA 95, 5145–5149. (10.1073/pnas.95.9.5145) 9560243 PMC20228

[43] Chee-Sanford JC, Aminov RI, Krapac IJ, Garrigues-Jeanjean N, Mackie RI 2001 Occurrence and diversity of tetracycline resistance genes in lagoons and groundwater underlying two swine production facilities. Appl. Environ. Microbiol. 67, 1494–1502. (10.1128/AEM.67.4.1494-1502.2001) 11282596 PMC92760

[44] Hanski I 1998 Metapopulation dynamics. Nature 396, 41–49. (10.1038/23876)

[45] Gonzalez A 1998 Metapopulation dynamics, abundance, and distribution in a microecosystem. Science (80-.) 281, 2045–2047. (10.1126/science.281.5385.2045) 9748167

[46] Ricketts TH 2004 Tropical forest fragments enhance pollinator activity in nearby coffee crops. Conserv. Biol. 18, 1262–1271. (10.1111/j.1523-1739.2004.00227.x)

[47] Duncan DH, Dorrough J, White M, Moxham C 2008 Blowing in the wind? Nutrient enrichment of remnant woodlands in an agricultural landscape. Landscape Ecol. 23, 107–119. (10.1007/s10980-007-9160-0)

[48] Blitzer EJ, Dormann CF, Holzschuh A, Klein AM, Rand TA, Tscharntke T 2012 Spillover of functionally important organisms between managed and natural habitats. Agric. Ecosyst. Environ. 146, 34–43. (10.1016/j.agee.2011.09.005)

[49] Rand TA, Tylianakis JM, Tscharntke T 2006 Spillover edge effects: the dispersal of agriculturally subsidized insect natural enemies into adjacent natural habitats. Ecol. Lett. 9, 603–614. (10.1111/j.1461-0248.2006.00911.x) 16643305

[50] Lindemann J, Upper CD 1985 Aerial dispersal of epiphytic bacteria over bean plants. Appl. Envir. Microbiol. 50, 1229–1232. 10.1128/aem.50.5.1229-1232.1985 PMC238730 16346928

[51] Dighton J, Jones HE, Robinson CH, Beckett J 1997 The role of abiotic factors, cultivation practices and soil fauna in the dispersal of genetically modified microorganisms in soils. Appl. Soil Ecol. 5, 109–131. (10.1016/S0929-1393(96)00137-0)

[52] Yao H, He Z, Wilson M, Campbell C 2000 Microbial biomass and community structure in a sequence of soils with increasing fertility and changing land use. Microb. Ecol. 40, 223–237. (10.1007/s002480000053) 11080380

[53] Bowers RM, McLetchie S, Knight R, Fierer N 2011 Spatial variability in airborne bacterial communities across land-use types and their relationship to the bacterial communities of potential source environments. ISME J. 5, 601–612. (10.1038/ismej.2010.167) 21048802 PMC3105744

[54] Pulliam HR 1988 Sources sinks and population regulation. Am. Nat. 132, 652–661. (10.1086/284880)

[55] Holt RD 1985 Population dynamics in two-patch environments: some anomalous consequences of an optimal habitat distribution. Theor. Popul. Biol. 28, 181–208. (10.1016/0040-5809(85)90027-9)

[56] Holt RD, Gaines MS 1992 Analysis of adaptation in heterogeneous landscapes: implications for the evolution of fundamental niches. Evol. Ecol. 6, 433–447. (10.1007/BF02270702)

[57] Urban MC, Skelly DK 2006 Evolving metacommunities: toward an evolutionary perspective on metacommunities. Ecology 87, 1616–1626. (10.1890/0012-9658(2006)87%5B1616:EMTAEP%5D2.0.CO;2) 16922313

[58] Roszak DB, Colwell RR 1987 Survival strategies of bacteria in the natural environment. Microbiol. Rev. 51, 365–379. 3312987 10.1128/mr.51.3.365-379.1987 PMC373117

[59] Perron GG, Gonzalez A, Buckling A 2007 Source–sink dynamics shape the evolution of antibiotic resistance and its pleiotropic fitness cost. Proc. R. Soc. B 274, 2351–2356. (10.1098/rspb.2007.0640) PMC2288555 17650474

[60] Gandon S 2002 Local adaptation and the geometry of host-parasite coevolution. Ecol. Lett. 5, 246–256. (10.1046/j.1461-0248.2002.00305.x)

[61] Thrall PH, Burdon JJ 2002 Evolution of gene-for-gene systems in metapopulations: the effect of spatial scale of host and pathogen dispersal. Plant Pathol. 51, 169–184. (10.1046/j.1365-3059.2002.00683.x)

[62] Heinemann JA, Ankenbauer RG, Amábile-Cuevas CF 2000 Do antibiotics maintain antibiotic resistance? Drug Discov. *Today* 5, 195–204. (10.1016/S1359-6446(00)01483-5) 10790263

[63] Rosenzweig ML 1987 Habitat selection as a source of biological diversity. Evol. Ecol. 1, 315–330. (10.1007/BF02071556)

[64] Holt RD 1996 Adaptive evolution in source-sink environments: direct and indirect effects of density-dependence on niche evolution. Oikos 75, 182–192. (10.2307/3546242)

[65] Bell T 2010 Experimental tests of the bacterial distance-decay relationship. ISME J. 4, 1357–1365. (10.1038/ismej.2010.77) 20535220

[66] Smith DJ, Timonen HJ, Jaffe DA, Griffin DW, Birmele MN, Perry KD, Ward PD, Roberts MS 2013 Intercontinental dispersal of bacteria and archaea by transpacific winds. Appl. Environ. Microbiol. 79, 1134–1139. (10.1128/AEM.03029-12) 23220959 PMC3568602

[67] Zinger L, Boetius A, Ramette A 2014 Bacterial taxa-area and distance-decay relationships in marine environments. Mol. Ecol. 23, 954–964. (10.1111/mec.12640) 24460915 PMC4230465

[68] Gregory TR 2005 Synergy between sequence and size in large-scale genomics. Nat. Rev. Genet. 6, 699–708. (10.1038/nrg1674) 16151375

[69] Konstantinidis KT, Tiedje JM 2004 Trends between gene content and genome size in prokaryotic species with larger genomes. Proc. Natl Acad. Sci. USA 101, 3160–3165. (10.1073/pnas.0308653100) 14973198 PMC365760

[70] Glaser P et al. 2001 Comparative genomics of *Listeria* species. Science 294, 849–852. (10.1126/science.1063447) 11679669

[71] Barberán A, Ramirez KS, Leff JW, Bradford MA, Wall DH, Fierer N 2014 Why are some microbes more ubiquitous than others? Predicting the habitat breadth of soil bacteria. Ecol. Lett. 17, 794–802. (10.1111/ele.12282) 24751288

[72] Lane N, Martin W 2010 The energetics of genome complexity. Nature 467, 929–934. (10.1038/nature09486) 20962839

[73] Mira A, Ochman H, Moran NA 2001 Deletional bias and the evolution of bacterial genomes. Trends Genet. 17, 589–596. (10.1016/S0168-9525(01)02447-7) 11585665

[74] Bouki C, Venieri D, Diamadopoulos E 2013 Detection and fate of antibiotic resistant bacteria in wastewater treatment plants: a review. Ecotoxicol. Environ. Saf. 91, 1–9. (10.1016/j.ecoenv.2013.01.016) 23414720

[75] Wang H, Zhang T, Wei G, Wu L, Wu J, Xu J 2014 Survival of *Escherichia coli* O157:H7 in soils under different land use types. Environ. Sci. Pollut. Res. Int. 21, 518–524. (10.1007/s11356-013-1938-9) 23812736

[76] Ramsayer J, Kaltz O, Hochberg ME 2013 Evolutionary rescue in populations of *Pseudomonas fluorescens* across an antibiotic gradient. Evol. Appl. 6, 608–616. (10.1111/eva.12046) 23789028 PMC3684742

[77] Andam CP, Carver SM, Berthrong ST 2015 Horizontal gene flow in managed ecosystems. Annu. Rev. Ecol. Evol. Syst. 46, 121–143. (10.1146/annurev-ecolsys-112414-054126)

[78] Mendes LW, Kuramae EE, Navarrete AA, van Veen JA, Tsai SM 2014 Taxonomical and functional microbial community selection in soybean rhizosphere. ISME J. 8, 1577–1587. (10.1038/ismej.2014.17) 24553468 PMC4817605

[79] Bell T, Newman JA, Silverman BW, Turner SL, Lilley AK 2005 The contribution of species richness and composition to bacterial services. Nature 436, 1157–1160. (10.1038/nature03891) 16121181

[80] Bell T, Gessner MO, Griffiths RI, McLaren JR, Morin PJ, Van Der Heijden M, Van Der Putten WH 2009 Microbial biodiversity and ecosystem functioning under controlled conditions and in the wild. In Biodiversity, ecosystem functioning, and human wellbeing: an ecological and economic perspective (eds Naeem S, Bunker DE, Hector A, Loreau M, Perrings C), pp. 121–133. Oxford, UK: Oxford University Press.

[81] Aanderud ZT, Jones SE, Fierer N, Lennon JT 2015 Resuscitation of the rare biosphere contributes to pulses of ecosystem activity. Front. Microbiol. 6, 24 (10.3389/fmicb.2015.00024) 25688238 PMC4311709

[82] Martiny JBH, Jones SE, Lennon JT, Martiny AC 2015 Microbiomes in light of traits: a phylogenetic perspective. Science 350, aac9323. (10.1126/science.aac9323) 26542581

[83] Strickland MS, Lauber C, Fierer N, Bradford MA 2009 Testing the functional significance of microbial community composition. Ecology 90, 441–451. (10.1890/08-0296.1) 19323228

[84] Yachi S, Loreau M 1999 Biodiversity and ecosystem productivity in a fluctuating environment: the insurance hypothesis. Proc. Natl Acad. Sci. USA 96, 1463–1468. (10.1073/pnas.96.4.1463) 9990046 PMC15485

[85] Wang S, Loreau M 2016 Biodiversity and ecosystem stability across scales in metacommunities. Ecol. Lett. 19, 510–518. (10.1111/ele.12582) 26918536 PMC4825858

