# Supplementary material for: Microbes in the Anthropocene: spillover of agriculturally selected bacteria and their impact on natural ecosystems
Source: Proc Biol Sci. 2016 Dec 14;283(1844):20160896. doi: 10.1098/rspb.2016.0896 (PMC5204138; doi:10.1098/rspb.2016.0896)
Supplement: Table S1: Summary of the hypotheses [file rspb20160896supp1.pdf]

Table S1. Hypotheses and predictions of the landscape-level impact of agriculture on soil microbial communities, and the consequences for ecosystem functioning in adjacent unmodified environments.

Hypotheses	Predictions
**Landscape-level impacts of agriculture on microbial communities**	
1. Agriculture selects for taxa and genotypes, which may differ from those in unmodified environments	• Community composition will differ between agriculture and unmodified environments.<br>o Within taxa: populations that experience intensive agriculture will have different genotypes to (or a subset of genotypes of) those in natural habitats.<br>o Among taxa: genotypic similarity will be higher due to horizontal transfer of agriculturally-selected genes.
2. Agriculturally selected genes will spill over into adjacent unmodified ecosystems	• Microbial cells will spill over from agricultural to natural habitats.<br>• Productivity differences among habitats will generate asymmetries in the rate of spillover, with greater net flow from the more productive habitat.
3. Landscape composition will mediate the impact of genetic spillover	• The extent to which agriculturally-selected traits become fixed in a given a taxon across the landscape will depend on the proportion of its total population in agricultural vs. non-agricultural habitats.<br>• The range over which spillover can drive introgression of genes into adjacent unmodified "sink" habitats will depend on the interplay between dispersal range of individual cells and the distance between source patches in the landscape.<br>• Some agriculturally selected traits will experience unforeseen fitness benefits in different contexts, which may allow them to spread further across the landscape. Such effects will depend on differences in selection regimes across habitat types.

4. The rate and range of dispersal will determine the extent of genetic spillover

- The ability of genes to become fixed regionally through the processes outlined in Hypotheses 1-3 will depend on dispersal dynamics. If dispersal is too high, we expect homogenous populations of generalists across the landscape. If dispersal is too low, there will be little opportunity for spillover to play a role.
- There will be a decline in the abundance of source bacterial populations, and of overall community similarity with distance from a source. The rate of decay should depend on the dispersal ability of the taxon, and should become steeper in heterogeneous landscapes.

5. There will be selection for large genomes when landscapes are heterogeneous and dispersal rates are high – the 'Swiss-army genome' hypothesis

- Bacteria with larger genomes have a greater functional repertoire, and will perform better in more heterogeneous landscapes, or when conditions are temporally variable.
- Increased genome size would be selected in heterogeneous landscapes with some intensified areas.
- If intensification spreads to comprise the majority of a landscape, selection will likely favour a specific set of phenotypic traits suited to agriculture, rather than genetic plasticity, resulting in a reduced genome size in highly intensified landscapes.

From process to pattern

6. There will be a halo of genetic differentiation surrounding intensive agriculture

- Spillover will generate a halo of species and/or genotypes surrounding the agricultural habitat in which they were selected. The size of this halo will be determined by the dispersal ability of each taxon and the heterogeneity of the landscape.
  - If the surrounding habitat has very different selection pressures: the halo will contain dead and dying cells, with living-cell densities that represent a balance of immigration and mortality rates.
  - If mortality is slow but immigration rate is high: the halo population with a negative growth rate could be maintained long enough by immigration to adapt to the novel conditions and achieve positive population growth. This would cause the halo to expand over time. An analogous process could occur with specific genotypes within a population.
  - If the taxa that spill over have traits that confer advantages outside agriculture: the halo would continually expand as they spread into the surroundings.

7. Landscape simplification will drive genetic homogenization both within and across taxa	• Expansion and intensification of agriculture will drive increased similarity of taxa across patches in the landscape, and increased genotypic similarity across taxa.
**Consequences of spillover for ecosystem functioning**	
8. The effects of spillover on community and genotypic composition will alter ecosystem functioning	• The above changes will also alter ecosystem functions performed by microbes. This may not necessarily be detectable as changes in taxonomic composition.
9. Microbial processes across the landscape will become increasingly variable due to loss of genetic insurance against environmental change / global stressors	• Aggregate ecosystem processes will be more variable in agriculturally-dominated landscapes.<br>• Within natural habitats, ecosystem functions will become more variable with increasing proximity to agricultural land, and with increasing intensity of that land.
